# Pancreatic cancer and fibrosis: Targeting metabolic reprogramming and crosstalk of cancer-associated fibroblasts in the tumor microenvironment

**DOI:** 10.3389/fimmu.2023.1152312

**Published:** 2023-03-22

**Authors:** Xin Li, Jianbo Zhou, Xue Wang, Chunxi Li, Zifan Ma, Qiaoling Wan, Fu Peng

**Affiliations:** Department of Pharmacology, Key Laboratory of Drug-Targeting and Drug Delivery System of the Education Ministry, Sichuan Engineering Laboratory for Plant-Sourced Drug and Sichuan Research Center for Drug Precision Industrial Technology, West China School of Pharmacy, Sichuan University, Chengdu, China

**Keywords:** pancreatic cancer, cancer-associated fibroblasts, fibrosis, metabolic reprogramming, crosstalk, heterogeneity

## Abstract

Pancreatic cancer is one of the most dangerous types of cancer today, notable for its low survival rate and fibrosis. Deciphering the cellular composition and intercellular interactions in the tumor microenvironment (TME) is a necessary prerequisite to combat pancreatic cancer with precision. Cancer-associated fibroblasts (CAFs), as major producers of extracellular matrix (ECM), play a key role in tumor progression. CAFs display significant heterogeneity and perform different roles in tumor progression. Tumor cells turn CAFs into their slaves by inducing their metabolic dysregulation, exacerbating fibrosis to acquire drug resistance and immune evasion. This article reviews the impact of metabolic reprogramming, effect of obesity and cellular crosstalk of CAFs and tumor cells on fibrosis and describes relevant therapies targeting the metabolic reprogramming.

## Introduction

Pancreatic cancer is one of the most aggressive types of cancer, being more common in developed countries and by low survival rates (1). As the main form of pancreatic cancer, pancreatic ductal adenocarcinoma (PDAC) has a discouraging prognosis, with a very low five-year survival rate (2). There is a correlation between lifestyle habits including smoking, alcohol consumption, and genetic and environmental factors and the onset of pancreatic cancer (1). Notably, the hormones, pro-angiogenic factors and pro-inflammatory cytokines secreted by obese tissues make obesity a risk factor for the occurrence of pancreatic cancer (3, 4). Diabetes associated with obesity and chronic pancreatitis also show a relevance to pancreatic cancer (5). Surgery is the treatment that has the potential to cure pancreatic cancer now, whilst chemotherapy, immunotherapy and targeted therapies have been demonstrated to help enhance the overall survival rate of patients (6–8).

Fibrosis driven by chronic inflammation occurs commonly in a variety of cancers, such as liver, pancreatic, and lung cancers (9–11). This formation of excessive intratumoral connective tissue is also referred to as desmoplasia by pathologists (12). Desmoplasia is one of the major pathological features and is intimately connected with its occurrence, progression and prognosis of pancreatic cancer. The desmoplastic reaction caused by inflammation gives pancreatic cancer an extraordinarily rich ECM (13). The fibrotic response in tumors is by the same mechanism as wound healing, being an excessive accumulation of ECM components and involving multiple cytokines and growth factors (14). ECM proteins are rich in composition, including fiber-forming proteins, glycoproteins, proteoglycans and matricellular proteins (15). The dense stroma leads to hypoxia in the tumor microenvironment and makes it difficult for chemotherapeutic agents to penetrate, thus imparting chemoresistance to pancreatic cancer (16).

TME of pancreatic cancer contains abundant stroma, blood vessels, and soluble proteins (17). Apart from cancer cells, three types of normal cells are found in the TME, namely stromal cells, fibroblasts, and immune cells (18). TME as a dynamic system has a changing composition and influences the progression of fibrosis in pancreatic cancer. Cancer-associated fibroblasts are extraordinarily abundant and secrete a range of extracellular matrix proteins, growth factors, and cytokines (19). CAFs crosstalk with tumor cells and immune cells and perform metabolic reprogramming to promote tumor development and fibrosis. In this review, we give a summary of current information about the heterogeneity of CAFs in pancreatic cancer cells, as well as updates on the metabolic reprogramming, crosstalk and therapies in the TME.

## Heterogeneity of CAFs

CAFs were initially thought to be homogeneous, but subsequent studies proved that CAFs varied in origin, expression, and function (20). Their typing remains incompletely elucidated, but existing work demonstrates that functionally distinct or even completely opposite subtypes exist. Öhlund et al. found pancreatic stellate cells (PSCs) were able to differentiate into two CAF subtypes, myofibroblastic CAFs (myCAFs) and inflammatory CAFs (iCAFs) in mouse PDAC (21). They differed significantly in spatial distribution and cytokine expression. myCAFs were distributed in the periglandular region at a closer distance from tumor cells, with high expression of α-smooth muscle actin (α-SMA) and low expression of interleukin (IL)-6, whereas iCAFs were distributed more distantly throughout the tumor, with low expression of α-SMA but high expression of cytokines such as IL-6, IL-11, and leukemia inhibitory factor (LIF) (21). This classification still has not reached the end point, as three subgroups of iCAFs were identified (22). Antigen-presenting CAFs (apCAFs) was identified in PDAC, named for its ability to express MHC class II molecules (23). A new CAF subtype with a highly activated metabolic state (meCAFs) was found in loose-type PDAC (24). Complement-secreting CAFs (csCAFs) were found in PDAC featuring a specific expression of complement components such as C3, C7, C1R/S, CFD, CFH, CFI (25). In the same study, Chen et al. defined PSCs as a subtype of CAF and found that PSCs dominated in PDAC stages I, II and III (25). The state of differentiation is reversible as iCAFs and myCAFs are able to convert into each other and apCAFs can also differentiate into myCAFs (21, 23). Modulation of transforming growth factor-β (TGF-β), IL-1/JAK/STAT signaling and hedgehog signaling impact on the differentiation of myCAFs and iCAFs (26, 27). Hypoxia within the TME probably converted fibroblasts to iCAFs (28). Neuzillet et al. proved the presence of at least four CAF subtypes in PDAC, which were featured by distinct mRNA expression profiles, with POSTN, MYH11, and PDPN as markers for three of the subtypes (29). PDPN-positive CAFs are molecularly similar to an iCAF subset, while POSTN-positive CAFs are not associated with the classical myCAF/iCAF classification (30). And these two subsets cooperate in the TME to induce the recruitment of monocytes/macrophages (30). It is worth mentioning that the identified subtypes of CAFs are not only present in pancreatic cancer, but also can be found in breast, ovarian and lung cancer models (31).

**Figure 1:**
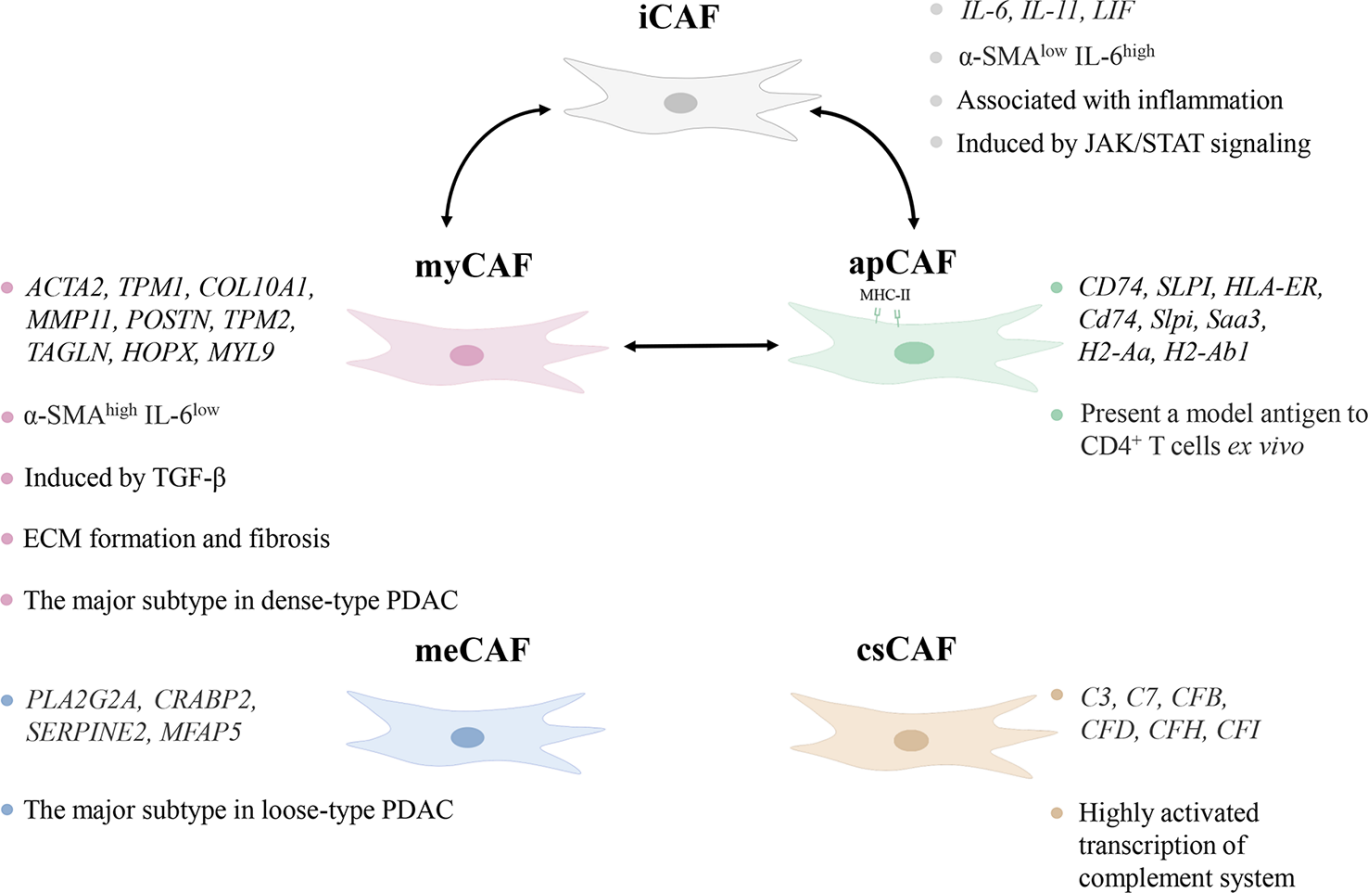
Characteristics of subtypes of cancer-associated fibroblasts (CAFs). The different subtypes of CAFs show heterogeneity in distribution, marker genes, and pathways in the tumor microenvironment. iCAF, inflammatory CAF; α-SMA, α-smooth muscle actin; IL-6, interleukin-6; myCAF, myofibroblastic CAF; TGF-β, transforming growth factor-β; ECM, extracellular matrix; PDAC, pancreatic ductal adenocarcinoma; apCAF, antigen-presenting CAF; meCAF, CAF with a highly activated metabolic state; csCAF complement-secreting CAF.

The major CAF subtypes show significant heterogeneity not only in phenotypes but also in function (**Figure 1**). The pathways enriched by myCAFs included ECM organization, and collagen formation were significantly upregulated, and its high α-SMA expression indicated its possible involvement in ECM formation and fibrosis (23). iCAFs highly expressed inflammatory cytokines, and up-regulated IFN-γ response, TNF/NF-κB, IL-2/STAT5, IL-6/JAK/STAT3, and the complement pathway (23). PDAC iCAFs were classified into different subsets, and OGN was a unique marker for one of those linked to a good prognosis (22). are correlated with a poorer prognosis, whereas another study linked higher abundance of iCAFs to a better prognosis (28, 30). This may result from the presence of different subgroups in iCAFs, but it also demonstrates that iCAFs simultaneously have tumor-promoting and inhibiting properties. circCUL2 regulated miR-203a-5p/MyD88/NF-κB/IL-6 axis to induce the production of iCAFs, which increased the secretion of IL-6, thereby promoting PDAC progression and immunosuppression (32). Huang et al. found that mesothelial cells were induced to differentiate into apCAFs by IL-1/NF-κB and TGF-β signaling (33). apCAFs promoted the transition of naive CD4^+^ T cells into regulatory T cells (Tregs), which means that it may be related to immunosuppression (33).

Although PSCs are generally considered to be the major precursor cells for CAFs within pancreatic cancer, a recent study indicated that PSCs produced only a small fraction of CAFs in PDAC (34). However, the promotion of fibrosis by PSCs remains an important component of pancreatic cancer progression. While activated PSCs are considered to be CAFs, for a clearer representation of the source, PSCs are described separately from CAFs in this review. PSCs were first identified in the intralobular and interlobular connective tissues of normal pancreas with lipid droplets containing vitamin A in 1982 (35). A study showed that vitamin A deficiency contributed to the transition of PSCs from a quiescent to the activated state (36). When injury or inflammation activates the quiescent PSCs, this vitamin A droplet disappears while the expression of collagen, fibronectin, laminin and α-SMA increases, and EMT production rises. The activation of PSCs is influenced by a variety of factors, including alcohol, diabetes, oxidative stress, cytokines, growth factors, etc. TGF-β1 is considered to be the main regulator, while platelet derived growth factor (PDGF), IL-6, IL-11, c-Jun N-terminal kinase (JNK) and extracellular signal-regulated kinase (ERK) are also implicated (37–40).

## Metabolic reprogramming in CAFs

**Figure 2:**
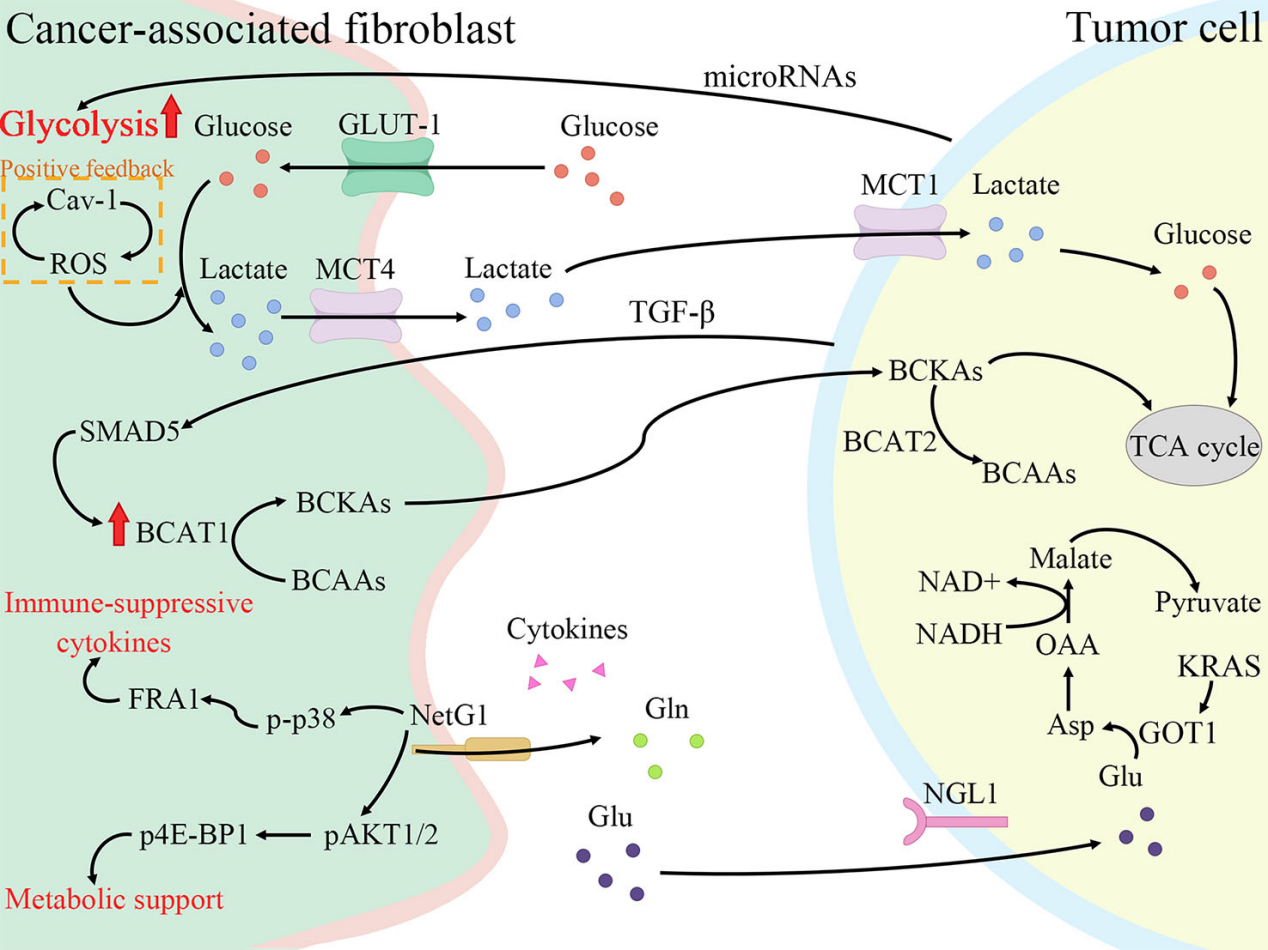
Cancer-associated fibroblasts (CAFs) promote fibrosis and tumor growth through metabolic reprogramming. CAFs increase glycolysis and glutamine secretion to supply lactate, branched-chain α-keto acids (BCKAs), glutamine, and cytokines to tumor cells. Meanwhile, tumor cells also secrete cytokines and microRNAs to regulate the metabolic reprogramming of CAFs to enable themselves to survive in a low-nutrient environment. Cav-1, Caveolin-1; ROS, reactive oxygen species; GLUT-1, glucose transporter-1; MCT4, monocarboxylate transporters 4; BCAT1, branched-chain amino acid transaminase 1; BCAAs, branched-chain amino acids; NetG1, Netrin G1; p-p38, phosphorylation of p38; FRA1, FOS-related antigen 1; pAKT1/2, phospho-AKT1/2; p4E-BP1, p4E-BP1; Gln, glutamine; Glu, glutamate; NGL1, Netrin-G ligand-1; TGF-β, transforming growth factor-β; MCT1, monocarboxylate transporters 1; BCAT2, branched-chain amino acid transaminase 2; TCA cycle, tricarboxylic acid cycle; GOT1, aspartate transaminase; Asp, aspartate; OAA, oxaloacetate.

Tumor cells still produce energy through less efficient aerobic glycolysis even under adequate oxygen, enhancing glucose transformation to pyruvate, termed the Warburg effect (41). However, this is not due to mitochondrial damage as originally envisioned by Warburg, but rather spontaneous metabolic reprogramming of tumor cells, where activation of a series of signaling factors and pathways leads to a switch from oxidative phosphorylation to glycolysis (42). Similar metabolic reprogramming exists in CAFs, and the Warburg effect is more obvious (43). Pancreatic cancer is one of the most severely hypoxic tumors as known, and hypoxia-inducible factors (HIFs) are the main regulators of hypoxia adaptation (44). Since the identification of HIF-1α in 1995, a wide range of roles of HIF-1 is continuously revealed in angiogenesis, cell metabolism, cell survival, and so forth (45, 46). In breast cancer, ROS production by cancer cells induces loss of Cav-1 in stromal cells, allowing CAFs to accumulate ROS and activate HIF-1α, consequently reprogramming CAFs and inducing autophagy (47, 48). The same alterations are shown in the PDAC model, where Cav-1 is lost in response to PSCs activation, correlating with stromal and cancer cells metabolic coupling (49). To conclude, HIF-1α connects oxidative stress and metabolic reprogramming of CAFs. Under such harsh conditions with hypoxia and low nutrition, there is metabolic crosstalk between CAFs with tumor cells and immune cells, all of which interact with each other to make TME a more habitable system (**Figure 2**).

### Glucose metabolism

Pavlides et al. proposed the reverse Warburg effect, elucidating that CAFs were able to perform glycolysis, producing pyruvate and lactate and making them available to cancer cells for use in the mitochondrial tricarboxylic acid (TCA) cycle (50). In other words, CAFs are captured by engaging with cancer cells and reprogrammed to a glycolytic phenotype. thereby supplying metabolic intermediates that enable cancer cells to compensatively generate energy *via* mitochondrial OXPHOS (51, 52). Glycolysis is the main metabolic mode of CAFs due to the increased expression of HIF-1α and monocarboxylate transporter (MCT) 4 (53). HIF-1α is a key cytokine that enables cells to adjust to hypoxic environments and undergo metabolic changes by promoting glycolysis through genes which encode glucose transporter proteins and enzymes of the glycolytic pathway (54). MCTs are passive transporter proteins that transport monocarboxylic acid ions and are highly expressed in tumors (55). MCT1 and MCT4 exhibit proton-coupled symport, with MCT4 generally involved in the export of lactate and MCT1 generally involved in the import of lactate (56). The expression of two glycolytic enzymes, lactate dehydrogenase A and pyruvate kinase M2, was found to be increased in CAFs (57). Furthermore, when pancreatic cancer cells were co-cultured with CAFs, MCT1 protein, succinate dehydrogenase and fumarate hydratase expression increased, demonstrating the metabolic coupling existing between CAFs and cancer cells (57). Positive feedback of Caveolin-1-ROS signaling prompted activation of PSCs and upregulated the expression of glycolytic enzymes, and the transporter protein MCT4, and downregulated the expression of OXPHOS enzymes and the transporter protein MCT1, while the protein expression in cancer cells was completely opposite (49). Moreover, MiR-21 promotes glucose uptake and lactate secretion by CAFs, indirectly enhancing pancreatic cancer cell invasion (58). Interestingly, CAFs also show heterogeneity in metabolic pathways, for iCAF had the highest metabolic activity and was more biased to glycolysis, whereas myCAF scored higher in OXPHOS than iCAF and apCAF (28).

### Amino acid metabolism

Glutamine, an amide of glutamate, is an essential origin of carbon and nitrogen in pancreatic cancer (59). Son et al. found that PDAC cells metabolized glutamine using a specific aspartate transaminase (glutamic-oxoacetic transaminase 1)-mediated pathway to produce biomass precursors and redox power (60). Glutamine also serves as an important energy source for CAFs and is metabolized and secreted into metabolites such as glutamate, α-ketoglutarate, aspartate and malate (53). Both Netrin G1 (NetG1) on NetG1^+^ CAFs and NetG1 ligand on tumor cells were highly expressed, resulting in the provision of glutamate/glutamine to tumor cells (61). NetG1 acts as a key regulator involved in ECM deposition, survival under low nutrient conditions and immunosuppression through the regulation of downstream pathways p38/FRA1 and AKT/4E-BP1 (61). PSCs increase glutamine synthetase expression by regulating the Wnt/β-catenin/TCF7 axis, thus promoting glutamine synthesis (62).

In addition to glutamine, alanine also acts as an important carbon source in the TCA of tumor cells. Tumor cells stimulate CAFs to catabolize metabolized proteins through autophagy to produce alanine and transaminate it to pyruvate (63). When glutamine is depleted, CAFs take up extracellular proteins through CaMKK2-AMPK-RAC1 signaling-dependent macropinocytosis and supply the produced amino acids to tumor cells (64). The macropinocytosis recovers CAFs to restore the production of collagen VI and fibronectin which is inhibited during glutamine depletion (64). In addition, the study also showed that protein-derived alanine was a secreted amino acid when serum albumin was cultured as a nutritional source for PSCs (64). It was demonstrated that pancreatic cancer cells and PSCs express SLC38A2 and SLC1A4 respectively to perform alanine exchange so as to meet the high alanine requirement of pancreatic cancer cells (65).

Furthermore, branched-chain amino acids (BCAAs), also known as leucine, isoleucine and valine, participate in metabolic reprogramming and crosstalk in CAFs and pancreatic cancer cells (66). Branched-chain amino acid transaminases (BCATs) can reversibly catalyze the transamination reaction of BCAAs to branched-chain α-keto acids (BCKAs) (67). TGF-β secreted by cancer cells upregulates BCAT1 activity by activating SMAD5 in CAFs, thereby increasing the secretion of BCKAs, which are supplied to cancer cells for BCAA synthesis (68).

### Lipid metabolism

Lipids form an important part of cellular biological membranes and building blocks, and are also involved in signaling and supplying energy (69). Multiple studies have demonstrated the existence of lipid metabolic reprogramming of CAFs in different cancer types, but regrettably there are not enough research in pancreatic cancer (70–72). PSCs undergo lipidomic remodeling upon activation, releasing lysophosphatidylcholine in large quantities to promote migration and proliferation of PDAC cells *via* the lysophosphatidylcholine-autotaxin-lysophosphatidic acid axis (73). Recently, a study found that activation of one PSC subpopulation is associated with elevated expression of lipoprotein-uptake very low-density lipoprotein receptor, which drives the expression of IL-33 (74). ROS-induced, endoplasmic reticulum stress-dependent increase in IL-33 expression mediates innate lymphoid type-2 cells activation, which induces proliferation and activation of PSCs, thereby stimulating pancreas fibrosis (74).

## Crosstalk: Complex communication between CAFs and tumor cells

CAFs take part in multiple stages of tumor progression, enabling bidirectional communication with other cells in the TME through intercellular contacts, secreted proteins and extracellular vesicles (75). Tumor cells signal CAFs to activate or secrete cytokines and matrix proteins, while CAFs promote drug resistance, proliferation, and migration of tumor cells. Here, we mainly summarize the signals from tumor cells that are significant for fibrosis.

### Extracellular vesicles

Extracellular vesicles are a form of intercellular communication that is currently of great interest. They are classified as prostasomes, apoptotic bodies, microvesicles and exosomes due to their size and origin (76). Exosomes contain a variety of nucleic acids(DNA, microRNA, lncRNA, circRNA), proteins, lipids and cytokines (77). We mention the ability of cancer cells to initiate metabolic reprogramming of CAFs, allowing them to provide nutrients to cancer cells. CD9, a specific exosome marker present on the surface of extracellular vesicles rich in annexin A6, enhances p38 mitogen-activated protein kinase signaling to induce PDAC cell migration (78, 79). Exosomes derived from PDAC cells expressing oncogenic KRAS mutants contain Survivin, imparting cell survival benefits to nearby CAFs (80). MiR-1246 and miR-1290 contained in pancreatic cancer cell-derived exosomes promote the expression of profibrogenic genes in PSCs (81).

### Secreted proteins

Mutated *KRAS* induces upregulation of plasminogen activator inhibitor-1 (PAI-1) in pancreatic cancer cells which induces PSCs activation *via* LRP-1/ERK/c-JUN pathway to promote immunosuppression and fibrosis (82). Meanwhile, PAI-1 expression was regulated by acyl-CoA synthetase long-chain 3, which may be associated with the regulation of TGF-β (83). High expression of PAI-1 not only promoted PSCs activation but also was associated with a high tumor infiltration of M2 macrophages (83). TGF-β1 represents a critical factor in the activation of PSCs. The secretion of TGF-β1 in pancreatic cancer cells is modulated by proteasome activator subunit 3-mediated activation protein-1, thus regulating the proliferation of PSCs (84). The induction of CAFs by TGF-β1 can be indirect, mediated through extracellular matrix proteins and growth factors such as PDGF, vascular endothelial growth factor (VEGF) and IL-6 (85). PDGF activates the hippo pathway and adds phosphorylation of yes-associated protein 1 in PSCs, and yes-associated protein 1 regulates the transcription of genes triggered by the TGF-β1/SMAD pathway, such as connective tissue growth factor and IL-6 (86). It has been shown that overexpression of galectin-1 stimulates the TGF-β1/Smad signaling pathway, with tissue inhibitor of metalloproteinase-1 (TIMP-1) expression increasing more than matrix metallopeptidase (MMP) 2, resulting in inhibition of ECM degradation and increased expression of fibronectin, collagen I and α-SMA (87). In addition, the paracrine of galectin-1 enhances the tumorigenic capacity of pancreatic epithelial cells (88). CXCL12/CXCR4 participates in the fibrotic process and the conversion of fibroblasts to myofibroblasts in multiple organs (89). Tumor-produced lactate causes epigenomic reprogramming when mesenchymal stem cells differentiate into CAFs (90). The increase of α-ketoglutarate causes C-X-C motif chemokine receptor 4 (CXCR4) promoter demethylation, leading to CXCR4 upregulation (90). Increase of special AT-rich sequence-binding protein 1 (SATB-1) expression in pancreatic cancer cells by CAFs through the SDF-1/CXCR4 axis further promotes CAFs activation (91). Furthermore, it has been established that tumor cells and CAFs crosstalk through nuclear factor KB (NF-κB) activated by paracrine-IL-1β. NF-κB activation by tumor-secreted IL-1β induces the expression of ESE3 in PSCs, then epithelium-specific E-twenty six factor 3 (ESE3) binds to the promoters of α-SMA, collagen-I and IL-1β, activating PSCs and promoting PDAC fibrosis (92). PDAC cells secrete IL-1β to activate IL-1 receptor-associated kinase 4 (IRAK4) in CAFs, forming an IL1β-IRAK4 feedforward circuitry that initiates fibrotic function in CAFs (93).

### Autophagy

Autophagy refers to a catabolic process to maintain intracellular homeostasis (94). But there is growing proof that autophagy takes part in the process of cellular secretion (95). Meanwhile, tumor cells are capable of secreting cytokines to induce autophagy in PSCs (63, 96). TGF-β1/Smad signaling-mediated autophagy promotes the conversion of fibroblasts to CAFs and facilitates their glycolysis (97). Activation of PSCs depends on autophagy, which is associated with the production of ECM and the secretion of IL-6 (96). CAFs conduct ribosomal RNA autophagy in a nuclear fragile X mental retardation-interacting protein 1 (NUFIP1)-dependent way, producing nucleosides available for PDAC cells under low nutrient conditions and initiating metabolic reprogramming (98). Collagen secretion can be facilitated by the mitophagy-regulated synthesis of proline in CAFs (99). In addition, a recent study found that PDAC cells generate lnc-FSD2-31:1 to promote the autophagy of CAFs *via* miR-4736, thereby inhibiting the activation of CAFs (100).

### Impact of crosstalk between CAFs and cancer cells on fibrosis

In TME, CAFs secrete large amounts of ECM proteins and remodeling enzymes to reorganize and stiffen the matrix (101). The main contribution of tumor cells to ECM deposition is the recruitment and activation of stromal cells. Multiple pathways of intercellular communication including protein secretion and extracellular vesicles enable pancreatic cancer cells to regulate the cellular activities of CAFs. Cancer cells are involved in the cross-linked sclerosis and degradation of ECM, aiding their invasion and migration from different aspects. Pancreatic cancer cells rely on multiple cytokines such as TGF-β, IL-1, sonic hedgehog (SHH), and microRNAs to activate CAFs and thus promote ECM stiffness (102). Meanwhile, pancreatic cancer cells also produce enzymes to promote matrix protein cross-linking in ECM such as lysyl oxidase-like protein 2 (LOXL2) (103). We summarize the cytokines and modes of action associated with fibrosis during the crosstalk between pancreatic cancer cells and CAFs (**Table 1**).

**Table 1:** Overview of the impact of crosstalk between CAFs and tumor cells on fibrosis.

Factor	Source	Mode of Action	Recipient cells	Functional Relevance	Reference
PAI-1	Pancreatic cancer cells	Paracrine	PSCs	Activates PSCs and promotes fibrosis	(82)
TGF-β1	Pancreatic cancer cells	Paracrine	PSCs	Promotes proliferation of PSCs	(84)
IL-1α	PDAC cells	Paracrine	PSCs	Promote ECM remodeling	(104)
IL-1β	PDAC cells	Paracrine	PSCs	promotes PSCs activation and expression of α-SMA, collagen I and IL-1β and activates CAFs to promote fibrosis	(92, 93)
PDGF	Pancreatic cancer cells	Paracrine	PSCs	Induces PSCs activation and promotes desmoplasia formation	(105)
SHH	Pancreatic cancer cells	Paracrine	PSCs	induces the expression of Gremlin 1 in PSCs	(106)
SATB-1	Pancreatic cancer cells	Paracrine	CAFs	Maintains CAFs identity and promotes the activation of CAFs	(91)
CXCL8	Pancreatic tumor cells	Paracrine	CAFs	maintains the survival of CAFs and further promotes FGF-2 production.	(107)
Oncogenic Kras-induced factors	PDAC cells	Paracrine	CAFs	Up-regulates the expression of CXCR2 and CXCR2 ligands in CAFs and induces the conversion of CAFs into iCAFs	(108)
miR-4736	PDAC cells	Extracellular vesicles	CAFs	Activates autophagy in CAFs, inhibits CAF activation and reduces fibrosis.	(100)
miR-155	Pancreatic cancer cells	Microvesicles	CAFs	Reprograms neighboring normal fibroblasts into CAFs	(109)
miR-1246, miR-1290 and miR-21-5p	Pancreatic cancer cells	Exosomes	PSCs	Promote the activation of PSCs and the production of collagen	(81)
Lin28B	Pancreatic cancer cells	Exosomes	Pancreatic cancer cells	Recruits PSCs	(110)
CCN2 or miR-21	PSCs	Exosomes	PSCs	Promotes collagen expression	(111)

PAI-1, plasminogen activator inhibitor-1; PSC, pancreatic stellate cells; TGF-β1, transforming growth factor-β1; IL, interleukin; ECM, extracellular matrix; α-SMA, α-smooth muscle actin; CAF, cancer-associated fibroblast; PDGF, platelet derived growth factor; SHH, sonic hedgehog; SATB-1, special AT-rich sequence-binding protein 1; FGF-2, fibroblast growth factor 2; PDAC, pancreatic ductal adenocarcinoma; iCAF, inflammatory CAF; Lin28B, lin-28 homolog B; CCN2, connective tissue growth factor.

## Obesity: An accomplice to pancreatic cancer fibrosis

Obesity is a critical independent risk factor for pancreatic cancer and is consistently associated with the development of pancreatic cancer. Obesity leads to hypertrophy and hyperplasia of adipocytes and causes chronic inflammation of the adipose tissue around or within the pancreas, which promotes tumor progression (112). And along with the advancement of pancreatic cancer stages, patients with pancreatic cancer experience adipose tissue loss as one of the manifestations of cachexia (113). Adipose tissue is divided into white, brown, and beige adipose tissue, while white adipose tissue is further classified into subcutaneous white adipose tissue and visceral white adipose tissue, with the latter playing a more pivotal role in the progression of pancreatic cancer (114). The cellular composition of white adipose tissue includes adipocytes, preadipocytes, immune cells, pericytes, endothelial cells, and multipotent stem cells (115). Some researches demonstrated the correlation of adipose tissue with fibroblast transformation and the formation and remodeling of ECM.

On the one hand, cells in adipose tissue have the ability to be reprogrammed into CAFs by pancreatic cancer cells. Adrenomedullin in the exosomes of pancreatic cancer cells promotes lipolysis in adipocytes (116). The lipolysis may explain the weight loss of the patients and represents a phenomenon of adipocytes dedifferentiation. Consequently, the dedifferentiation possibly connects the cachexia with fibroplasia in pancreatic cancer. When co-cultured with pancreatic cancer cells, 3T3-L1 adipocytes dedifferentiated to fibroblast-like cells, losing lipid droplets and expressing S100A4, MMP11, collagen I and α-SMA (117, 118). The reprogramming is closely correlated with WNT5a signaling (119). Adipose tissue-derived stromal cells can also be recruited to extrapancreatic invasive lesions and differentiate into CAFs, producing a more rigid ECM (120). Mucin 5AC secreted by pancreatic cancer cells recruits mesenchymal stem cells (MSCs) to a-SMA^+^ CAFs (121). Activin A produced by PDAC cells was found to be associated with the loss of adipose tissue and the promotion of fibrosis, with an induction of trans-differentiation of white adipocytes into fibrotic cells (122). On the other hand, adipocytes mediate fibrosis by crosstalk with neighboring cells *via* paracrine secretion. Adipocytes secrete IL-1β to recruit neutrophils, thereby enhancing the activation of PSCs (123).

## The therapy progress of reprogramming

**Figure 3:**
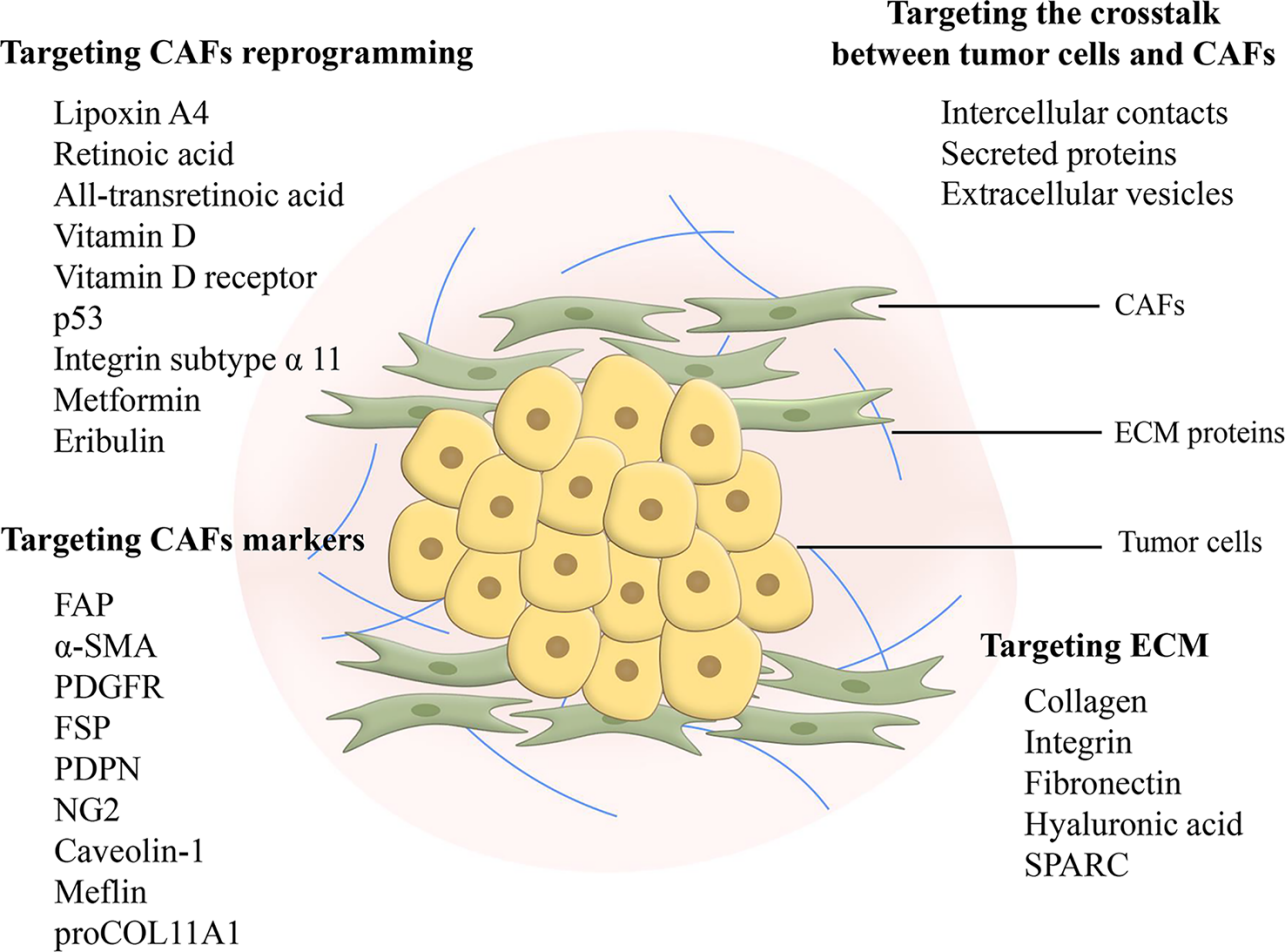
Feasible design ideas for targeting fibrosis. Targeting CAFs in metabolic reprogramming and signaling communication with cancer cells is considered as a promising therapeutic modality. Targeting markers of CAFs can inhibit CAFs, but there is a problem of non-specificity and further search and research is still needed. Targeting ECM elimination requires limitations, as sustained defibrosis implies enhanced invasion. In addition, ECM protein interactions may become an emerging therapeutic target. CAF, cancer-associated fibroblast; FAP, fibroblast activation protein; α-SMA, α-smooth muscle actin; PDGFR, platelet-derived growth factor receptor; FSP, fibroblast-specific protein; PDPN, podoplanin; NG2, nerve/glial antigen 2; ECM, extracellular matrix; SPARC, secreted protein acidic and rich in cysteine.

Ideas for targeting CAFs as therapeutic targets in pancreatic cancer for clinical benefit are diverse including depletion of CAFs, reprogramming CAFs to make them normal, and blocking signals from CAFs **(** **Figure 3**) (124). However, studies concerning the depletion of CAFs demonstrated that this treatment could lead to the exact opposite of what was expected, a facilitation of tumor progression (125, 126). Reprogramming CAFs to the stationary case is currently considered a feasible approach. It has been preliminarily demonstrated to be viable to normalize CAFs through endogenous substances, gene regulation, agents and intercellular interactions. Lipoxin A4 reversed the activation of PSCs to CAFs for matrix reprogramming, with decreased expression of α-SMA and collagen I (127). The increase of retinoic acid was able to inhibit CAFs and reduce the expression of α-SMA and FAP (128). Zhao et al. constructed a targeted drug delivery system based on red blood cells vesicles partial protection to deliver retinoic acid to CAFs to disrupt the Golgi apparatus and thereby inhibit the secretion of proteins such as MMP2, MMP9 and CCL2 (129). In addition, all-transretinoic acid inactivated PSCs by inhibiting Yes-associated protein 1 (YAP1) (130). Vitamin D and its receptor were involved in stromal reprogramming as well by inactivating CAF/PSC (131, 132). The activation of p53 could directly induce the accumulation of cytoplasmic lipid droplets in PSCs, thus effectively reprogramming PSCs to a quiescent state (133). Integrin subtype α 11 was also considered as a viable target for controlling the phenotype and activation of PSCs (134). Several studies have shown that metformin can reprogram PSCs to improve desmoplasia (135–137). Metformin inhibited TGF-β1 secretion by activating AMPK in pancreatic cancer cells, leading to blocking the activation of PSCs (136). In addition, eribulin also showed potential for normalizing CAFs due to its simulation of TGF-β downregulation (138). Mechanical regulation of intercellular interactions such as N-cadherin and N-cadherin ligand linkages could reprogram PSCs to a stationary state, however not in all cases, as this reprogramming was associated with mechanical dosing (139). Unfortunately, studies on the regulation of the metabolism of CAFs are scarce, because the mechanism of metabolic reprogramming of CAFs is still not entirely clarified. Chen et al. designed a liposome carrying hydroxychloroquine and paclitaxel to target autophagy in CAFs, with advantages for crosstalk and fibrosis inhibition (140). A biomimetic nanocarrier was devised to disrupt metabolic crosstalk by blocking lactate production in both CAFs and cancer cells (141).

## Discussion

CAF synthesizes, remodels and crosslinks ECM to increase stiffness leading to the generation of a dense fibrotic tumor stroma (101). CAFs act in pancreatic cancer progression as an essential component of the stroma. Five subtypes of CAFs have been identified so far, namely myCAFs, iCAFs, apCAFs, meCAFs and csCAFs, showing differences in expression and function in pancreatic cancer. This is still not the endpoint of the classification of CAFs, and the subtypes may contain subpopulations. As we mentioned before, different subpopulations of iCAFs may have opposite effects on tumor development. It implies that therapies targeting CAFs need more specific biomarkers. Different subtypes of CAFs relate to the discrepant prognosis of pancreatic cancer patients (24, 28).

Cells in the TME interact with each other to co-construct a microenvironment suitable for tumor survival. CAFs conduct metabolic reprogramming to provide available metabolites to tumor cells (**Table 2**). Oxidative stressed-driven metabolic changes in CAFs are known as the reverse Warburg effect, manifested by glycolysis as the main mode of metabolism and increased utilization of glutamine. Multiple forms of crosstalk including direct contact, extracellular vesicles, paracrine and autophagy-dependent secretion between tumor cells and CAFs activate CAFs for fibrosis on the one hand and enhance tumor cells proliferation and migration on the other. Cellular communication also exists between adipocytes and other cells in the TME. Lipolysis occurs when adipocytes dedifferentiate into CAFs, which perhaps partially explains both the cachexia and desmoplasia.

**Table 2:** Summary of CAFs metabolic reprogramming.

Metabolism	Regulated factors		Effects on metabolism	Origin	Reference
	Types	Effects			
Glycolysis	HIF-1α and MCT4	Up-regulation	Increase lactate production and glucose intake	CAFs	(53)
	HK-2, PFKP and PKM2	Up-regulation		PSCs	(49)
	TOMM20 and NQO1	Down-regulation		PSCs
	LDHA and PKM2	Up-regulation		CAFs	(57)
	miR-21	Up-regulation		CAFs	(58)
Glutamine metabolism	GLS1 and GLUD1	Up-regulation	Promote the production of glutamate and α-ketoglutarate	CAFs	(53)
	NetG1	Up-regulation	Promotes the secretion of glutamine and glutamate	CAFs	(61)
	glutamine synthetase	Up-regulation	Promotes the secretion of glutamine	PSCs	(142)
Alanine metabolism	SLC1A4	Up-regulation	Maintains alanine concentration in TME	PSCs	(65)
Branched-chain amino acid metabolism	BCAT1	Up-regulation	Increases the secretion of BCKAs	CAFs	(68)
Lipid metabolism	FABP4, PLIN1 and PLIN2	Down-regulation	Exhibit the remodeling of the intracellular lipidome	PSCs	(73)
	VLDLR	Up-regulation	Promotes lipoprotein uptake	PSCs	(74)

HIF-1α, hypoxia-inducible factor-1α; MCT4, monocarboxylate transporter 4; CAF, cancer-associated fibroblast; HK-2, hexokinase 2; PFKP, 6-phosphofructokinase; PKM2, pyruvate kinase isozyme type M2; PSC, pancreatic stellate cell; TOMM20, pyruvate kinase isozyme type M2; NQO1, NAD(P)H dehydrogenase [quinone] 1; LDHA, lactate dehydrogenase A; GLS1, glutaminase 1; GLUD1, glutamate dehydrogenase 1; NetG1, Netrin G1; TME, tumor microenvironment; BCAT1, branched-chain amino acid transaminase 1; BCKA, branched-chain α-keto acid; FABP4, fatty acid binding protein 4; PLIN1, perilipin 1; PLIN2, perilipin 2; VLDLR, very-low-density lipoprotein receptor.

Experimentally, depletion of CAFs proved to be an infeasible treatment. Reprogramming CAFs to a normal state or blocking signaling may be promising ways to target pancreatic cancer fibrosis. In conclusion, CAFs are important targets to explain fibrosis and drug resistance in pancreatic cancer, but further studies on the heterogeneity of CAFs and the mechanisms of crosstalk are still needed to provide more basis for targeting CAFs for therapy.

## Author contributions

The manuscript was written by XL, JZ, XW, ZM, CL and QW. XL and XW designed and made the figures. FP and JZ revised the manuscript. FP supported the study. All authors contributed to the article and approved the submitted version.
